# Does language dominance affect cognitive performance in bilinguals? Lifespan evidence from preschoolers through older adults on card sorting, Simon, and metalinguistic tasks

**DOI:** 10.3389/fpsyg.2014.00011

**Published:** 2014-02-05

**Authors:** Virginia C. Mueller Gathercole, Enlli M. Thomas, Ivan Kennedy, Cynog Prys, Nia Young, Nestor Viñas Guasch, Emily J. Roberts, Emma K. Hughes, Leah Jones

**Affiliations:** ^1^Linguistics Program, English Department, Florida International UniversityMiami, FL, USA; ^2^School of Education, Bangor UniversityBangor, UK; ^3^School of Social Sciences, Bangor UniversityBangor, UK; ^4^State Key Laboratory of Brain and Cognitive Sciences, University of Hong KongHong Kong, China; ^5^School of Psychology, Bangor UniversityBangor, UK

**Keywords:** executive function, bilingual children, language balance, language dominance, dimensional change card sort task, Simon task, metalinguistic task, Welsh bilinguals

## Abstract

This study explores the extent to which a bilingual advantage can be observed for three tasks in an established population of fully fluent bilinguals from childhood through adulthood. Welsh-English simultaneous and early sequential bilinguals, as well as English monolinguals, aged 3 years through older adults, were tested on three sets of cognitive and executive function tasks. Bilinguals were Welsh-dominant, balanced, or English-dominant, with only Welsh, Welsh and English, or only English at home. Card sorting, Simon, and a metalinguistic judgment task (650, 557, and 354 participants, respectively) reveal little support for a bilingual advantage, either in relation to control or globally. Primarily there is no difference in performance across groups, but there is occasionally better performance by monolinguals or persons dominant in the language being tested, and in one case-in one condition and in one age group-lower performance by the monolinguals. The lack of evidence for a bilingual advantage in these simultaneous and early sequential bilinguals suggests the need for much closer scrutiny of what type of bilingual might demonstrate the reported effects, under what conditions, and why.

## Introduction

The question of bilinguals' linguistic and cognitive abilities relative to those of their monolingual counterparts has been the subject of intense study and scrutiny over the last century. Debates have examined children's and adults' capacities in a number of linguistic and cognitive realms. Early studies had mixed results concerning whether bilingualism was seen to have negative or positive effects on cognition, but many studies were flawed in that they did not control, e.g., for socioeconomic or cultural differences (Hakuta, 1986; Cummins, 1992; Oller and Pearson, 2002; Genesee et al., 2004). Recently, more controlled studies have indicated a complex picture. It is clear that in some ways, bilinguals' knowledge of certain aspects of their languages—in particular in lexical, morphological and syntactic realms—is affected by amount of exposure, so their abilities may show initial delays relative to those of their monolingual cohorts (Ben-Zeev, 1977; Umbel et al., 1992; Pearson et al., 1993, 1995; Pearson and Fernández, 1994; Gathercole, 2002a,b,c, 2007a,b; Gathercole and Hoff, 2007; Thomas and Gathercole, 2007; Gathercole and Thomas, 2009).

At the same time, bilinguals have been reported to show an advantage over their monolingual peers in the realms of metalinguistic abilities (Bialystok, 1993) and cognitive abilities related to executive function (Zelazo and Müller, 2002; Blair et al., 2005), involving selective attention, inhibition of attention, and switching attention in tasks with competing and misleading cues (Johnson, 1991; Bialystok et al., 2004; Hernandez Pardo et al., 2008). In these tasks, a high degree of cognitive control (Bialystok and Ryan, 1985) must be maintained, whether to inhibit irrelevant cues or to “detach” the verbal message from its reference (e.g., separate the linguistic form from its meaning). Successful completion entails ignoring conflicting or extraneous information. Bialystok (1993, 1999, 2001) argues that bilinguals have an advantage here because from the beginning of their use of two languages, bilinguals must control and suppress the use of one language while using the other (see also Cummins, 1976; Hakuta, 1986; Johnson, 1991; Green, 1998). This is purported to lead to more fully developed neurological mechanisms for controlling such attention, referred to as “executive function,” which is relevant to the types of non-linguistic tasks mentioned (Bialystok and Ryan, 1985; Zelazo and Müller, 2002; Blair et al., 2005).

The advantage of bilinguals is reported, e.g., for the Stroop (1935) task, in which individuals are shown a color word written in a font of a color different from the color named by the word (e.g., *green* written in a red font) and are asked to name the color of the font, not read the word. In one study, Bialystok et al. (2008) found that younger and older adults showed a greater Stroop effect (i.e., a greater cost in this condition than in non-conflict conditions) among monolinguals than bilinguals. They reasoned that monolinguals may show a greater Stroop effect because of their greater automaticity of reading. But even the performance of a group of monolinguals who were slower readers showed a greater Stroop effect than a group of bilinguals who were fast readers. (The important role that language and literacy abilities play in monolinguals' and bilinguals' performance is discussed further below).

In another task, the Simon task (Martin and Bialystok, 2003; Bialystok et al., 2004), participants are shown colored stimuli on the left or right side of a computer, and they are asked to press a key—one on the left or one on the right—according to the color of the stimulus on the computer. “Incongruent” trials, in which the stimulus and the correct key are on opposite sides of the computer, take more time (the “Simon effect”) than “congruent” trials, in which the stimulus and the key are on the same side. Bilingual 4-year-olds show less of a Simon effect, and indeed also an advantage in the “congruent” cases, than monolinguals. (See below regarding the possibility of a global advantage in bilinguals.)

A third type of task is the “dimensional change card sort task” (Frye et al., 1995; Zelazo et al., 1996; Bialystok, 1999). In this task, participants are shown two target cards, one representing, e.g., a circle of one color (blue) and the other a square of another color (red), and then several other cards also showing opposite-colored circles and squares. The child is asked first to sort the items according to one dimension (e.g., color), and then to sort according to the other (shape). Bilingual children respond more accurately and more quickly than monolinguals on the second sort (Bialystok, 1999).

The bilingual advantage in control tasks is argued to also lead to superior performance by bilinguals in certain conditions of yet another type of task, a metalinguistic judgment task. Work conducted by Bialystok and colleagues (Bialystok, 1986, 1988; Barac and Bialystok, 2012) has argued that, while general performance on grammaticality judgment tasks, especially in judging ungrammatical sentences (e.g., “Why the dog is barking so loudly?”), seems to be related to level of knowledge of the language, there is one condition in which bilinguals are said to outperform monolinguals across the board, regardless of their level of bilingualism. That is on grammatically correct, but anomalous sentences, such as “Why is the cat barking so loudly?” (Bialystok, 1988: p. 565). The superior performance by bilinguals in this condition is attributed directly to superior executive control of bilinguals: This is “because attention normally directed to the meaning of the sentence [has] to be intentionally suppressed. Thus, the judgment require[s] high levels of control” (Bialystok, 1988, p. 565). Thus, “[o]n these problems, bilingual children consistently outperform monolingual children (Bialystok, 1986; Cromdal, 1999)” (Hermanto et al., 2012, p. 133).

Despite the many studies documenting such a cognitive advantage in bilinguals, some research has challenged the generality of the effect. Some have questioned the source of the effect, some have argued for better control over the choice of bilingual participants (e.g., Namazi and Thordardottir, 2010), and some have reported sporadic effects (Hilchey and Klein, 2011) or no bilingual effect (Paap and Greenberg, 2013), and there may be some with null effects that have not reached publication: Adesope et al. (2010) caution that there may be a “publication bias” against studies showing null or negative effects.

Yang and Lust (2004), for example, found no difference between monolingual and bilingual children's performance on a dimensional change card sort task but an advantage of bilinguals in an attentional network test, including a flanker task. They note that their monolinguals performed better on a vocabulary task, and so they suggest that language ability may have contributed to the lack of an effect for the card sort tasks; furthermore, their study controlled for the L1 languages of their participants, whereas many such studies pool participants from a variety of linguistic backgrounds and levels of proficiency. Variations in the first language backgrounds could have an effect on performance: Yang and Lust (2007) reported that children learning Korean and Chinese showed better performance on executive function tasks than those learning Spanish, regardless of linguality status (monolingual vs. bilingual), and in a systematic review of the literature, Adesope et al. (2010) reported significant differences in performance across distinct geographical and language groups, especially in relation to metalinguistic abilities.

Rosselli et al. (2002) also controlled for language background in a study of Spanish-English bilinguals' and monolinguals' performance on Stroop tasks. They found that bilinguals' performance was on the whole equivalent to monolinguals'. The one exception was that when asked to respond in English, bilinguals were generally slower than monolinguals, and Spanish-dominant bilinguals were slower than both English-dominant and balanced bilinguals. They suggest that the color naming effects may be related to vocabulary size. (See also Sumiya and Healy, 2004).

Similarly, in Chen and Ho (1986), Chinese L1-English L2 speakers in grade 2 through college performed Stroop tasks in Chinese and English; in some cases the language of the stimulus was the same as the language of the response, and in some different. The general finding was that within-language responding created greater interference than between-language responding, except for the youngest children. For these children, responses in English took longer with Chinese stimuli than with English stimuli. Since the younger children were less proficient in English, these results suggest that proficiency plays a role in the presence of the Stroop effect: the greater the proficiency, the more likely the within-language interference. Paap and Greenberg (2013) similarly report a lack of a bilingual advantage on a series of tasks when only highly proficient bilinguals are compared with monolinguals.

Socio-economic level might also contribute to results (Morton and Harper, 2007); monolingual and bilingual populations tested in some studies may have come from distinct socio-economic backgrounds (e.g., monolinguals from the general local population, bilinguals from L2 immigrants seeking higher education or from high SES academic parents choosing bilingual education for their children), and the effects of bilingualism may be more pronounced at some SES levels than at others (Woodard and Rodman, 2007). Hilchey and Klein (2011) point out the vast differences in sociocultural backgrounds of the bilingual vs. monolinguals in a series of studies, and caution that there may be many such “hidden factors” other than linguality *per se* that lead to differences in performance. This point regarding SES is important because of recent work (Neville, 2009) indicating profound cognitive and neurological effects of SES level on attention in children. In a recent study, Paap and Greenberg (2013) tested monolingual and bilingual college students in California on a Simon task and a flanker task, and controlled for parental education. They found no significant difference between monolingual and bilinguals on either task. In another study (Duñabeitia et al., 2013), monolingual and bilingual children in the Basque country were carefully matched on a variety of skills (reading, arithmetic, verbal, IQ, etc.), and were tested for performance on a classic verbal Stroop task and a numerical Stroop task. These researchers consistently failed to find any significant difference in performance between the monolinguals and bilinguals.

Thus, the source and generality of experimental effects in bilinguals vs. monolinguals is not always clear, demonstrating the need for more well-controlled studies. Hilchey and Klein (2011) suggest:

When these factors are not well controlled, a primary concern is that some of them might contribute or lead directly to what would appear to be bilingual processing advantages, and indeed, concerns of this sort have permeated the bilingualism literature. (p. 642).

The contributions of degree of proficiency in the language, SES factors, general cognitive abilities, age, and gender (and interactions between these) are still little understood in relation to bilinguals' and monolinguals' performance. Even the role of language dominance in the bilingual's performance is still unclear—it is not known to what extent various levels of language dominance might affect the cognitive benefits of bilingualism (Bialystok, 1988; Bialystok et al., 2004).

Furthermore, some have argued for a general cognitive advantage in bilinguals, not an advantage for inhibitory control (Hilchey and Klein, 2011). Hilchey and Klein (2011) review the evidence to date for a bilingual inhibitory control advantage (BICA), and conclude that there is little support for this position. In contrast, they argue, the evidence supports a more global bilingual executive processing advantage (BEPA) that leads to superior performance not only in conflict conditions (incongruent trials) but also in non-conflict conditions (congruent trials), particularly for RTs. They propose an alternative account, drawing on a conflict-monitoring system, to explain this global advantage; a similar account has been proposed by Costa et al. (2009). Paap and Greenberg (2013), like Hilchey and Klein (2011) failed to find in a series of studies any Group × Condition interactions revealing superior performance of bilinguals on conflict conditions. However, in contrast to Hilchey and Klein, Paap and Greenberg report no global advantages for bilinguals on their tasks. In fact, they argue, it is important that there is not a consistent pattern of performance by individuals across tasks: The failure to find consistent bilingual advantages across distinct components of executive processing challenges any theory of a unified account for results, even when a bilingual advantage is observed.

Adesope et al. (2010) note that often studies do not give clear information on the type of bilingual tested. In many studies, bilinguals are chosen as “balanced” on the basis of the fact that they have spoken both of their languages on a daily basis throughout their lives, but bilinguals lie on continua of dominance (Hakuta, 1987). Balanced bilinguals are not necessarily the same as those who use both of their languages on a daily basis (Grosjean, 1994; Grosjean and Li, 2003), and fully balanced bilinguals are quite rare (Hakuta, 1987).

Ultimately, the extent to which each factor contributes to performance is not well-understood. As Hilchey and Klein (2011, p. 643) say, “The onus is now on current investigative work to ensure that these factors are not influencing experimental outcomes.”

The goal of the present study was to test performance on a series of executive function tasks in a carefully controlled study on bilinguals and monolinguals who grew up in the same context. The data come from Welsh-English bilinguals living in North West Wales. This group can provide insight into the effects of bilingualism in individuals who grow up as bilinguals—either as 2L1 simultaneous bilinguals or as early sequential bilinguals who begin the second language by age 4 at the latest—in comparison with monolinguals who are from the same sociocultural background.

In this study, we strictly divide the bilingual participants, first, according to the languages that their parents speak to them in the home—only Welsh at home (OWH), Welsh and English (WEH), or only English (OEH). In our work on children's acquisition of Welsh (Gathercole et al., 2001, 2005; Gathercole and Thomas, 2005; Thomas and Gathercole, 2005; Thomas et al., 2013) and on bilingual language transmission in Welsh homes (Gathercole, 2007a), we have found consistent differences across groups in the timing of acquisition or specific abilities in Welsh vs. English. The greater the exposure to Welsh, the earlier the child develops Welsh structures and vocabulary; the greater the exposure to English, the earlier the development of English forms; children who have equal exposure fall between these two groups (see Gathercole and Hoff, 2007; Gathercole, 2010; Thomas and Mayr, 2010).

The determination of relative “dominance” (where dominance is defined according to relative abilities in the two languages) across the three home language groups is not unproblematic, however. Typically, at initial stages, OWH children can be considered the most Welsh-dominant of the three types, WEH the most balanced, and OEH the most English-dominant. By the teen years, the differences across the groups become indistinguishable in English, but the OWH group still surpasses the others in Welsh. So OWH speakers may be considered the most balanced at older ages (see Gathercole et al., 2013, 2014).

In a previous study (Gathercole et al., 2010), we administered tapping tasks and a Stroop task to primary school aged children and teenagers. We examined the contributions of home language, language abilities and usage, general cognitive performance, and socioeconomic level to children's performance on these two tasks. Results revealed a complex picture of their contributions. In the case of the tapping task, in which there was a copy condition and a switch condition, the analyses showed an overall advantage at primary age in the OWH and OEH children, with monolingual English (“MonE”) children performing least well, but there was no evidence of an advantage of any group in just a switch task, or on difference scores. By teen age, the OWH and WEH children showed better performance than the MonE and OEH children. The follow-up analyses indicated, further, a high degree of association between tapping performance and general number abilities and pattern discrimination abilities, and supported the initial results showing superior performance among those bilinguals who began Welsh earlier and English later and who speak a high percentage of Welsh.

In the Stroop task, participants were tested either in Welsh or in English on four conditions, one of which was the classic Stroop condition. Analyses showed no home language effect in Welsh at either age. For English, by the teen years, there was no home language effect, including no difference between the monolinguals and the bilinguals. At the younger age, the WEH children showed an advantage over the OWH and MonE participants, but the OWH children showed inferior performance on a control condition in which they had to retrieve the color name from their lexical store. This supports the position of a bilingual advantage, here in the WEH children, but also important contributions of automaticity related to literacy. Follow-up analyses also confirmed important contributions to performance of balanced use of the two languages, of SES, of overall cognitive abilities, and of general linguistic knowledge, as measured by vocabulary scores.

In order to document more fully where and when a bilingual advantage might occur in this type of bilingual population—fully bilingual participants who grew up or are growing up as simultaneous bilinguals or early sequential bilinguals, we administered a series of tasks on Welsh-English bilinguals from seven age groups, across the lifespan. The following experiments report on card sorting tasks, Simon tasks, and a metalinguistic judgment task, providing further evidence on the influence of bilingualism on tasks related to executive function.

## General research method

Participants in seven age groups (from 3 years of age through over 60 years of age) were administered several executive function tasks, including the card sorting, Simon, and metalinguistic tasks to be reported here. Participants were also administered, when possible, vocabulary tests in English (Dunn et al., 1982) and Welsh, tests of receptive grammatical knowledge in Welsh and English, and tests of general (non-executive function) cognitive abilities (McCarthy, 1972; Raven et al., 1983). Parents or the participants themselves filled out an extensive background questionnaire that included information on language use in the home and at school, parental language background, and parental education and professions. (We will report on effects involving non-language factors in a later study).

We predicted that the overall findings would be consistent with superior performance by bilinguals, especially the balanced bilinguals, over monolinguals. In the case of the card sorting tasks, the prediction was that this advantage would be observable in greater accuracy or faster reaction times of the (balanced?) bilinguals over the monolinguals in the tasks involving a switch of the parameters on which to base the sort; in the case of the Simon tasks, the prediction was that (balanced?) bilinguals would show an advantage in the conflict condition. For the metalinguistic task, the prediction was that (balanced?) bilinguals would show a particular advantage in the condition that involved grammatical but anomalous sentences.

### Participants

A total of 650 children and adults participated in the card sort tasks, 557 in the Simon tasks, and 354 in the metalinguistic task. With the exception of the metalinguistic task (which was not administered to the preschoolers) participants took part in all studies. Differences in numbers are due to attrition. Participants were recruited through schools in and around North Wales, bilinguals from Gwynedd, Denbigh, and Conwy counties, and monolinguals from the Chester area, just across the Welsh border into England. Informed consent was obtained from participants or parents of participants. Across the tasks, participants fell into 7 age categories and four major home-language groups. Children came from 5 different age groups, around 3, 4, and 5 years of age, 8 years of age (henceforward “primary schoolers”), and 15 years of age (henceforward “teens” or “teenagers”); adults came from two groups, younger adults and older adults. (Exact ages will be reported with each task). All age groups performed (different versions of) a card sort task and a Simon task, and those of primary school age and above a metalinguistic task.

On the basis of the background questionnaires, participants were classified as either monolinguals (“MonE”) or bilinguals coming from homes in which only Welsh was spoken (“OWH”), both Welsh and English were spoken (“WEH”), or only English was spoken (“OEH”)^1^.

## Card sort

### Methods

#### Participants

The distribution of participants for the card sort tasks was as shown in Table 1. Mean ages are shown in Appendix A.

**Table 1:** **Participants, card sorting tasks**.

**Age group**	**MonE**	**OEH**	**WEH**	**OWH**	**TOT**
3	14	20	16	21	71
4	29	19	18	18	84
5	19	21	21	19	80
Primary schoolers	25	22	20	29	96
Teens	20	24	26	34	104
Younger adults	28	20	28	30	106
Older adults	23	24	19	31	97
Total	161	151	150	188	650

#### Stimuli and procedure

Three types of card sort task were given, according to participants' age groups. The school age children and adults were provided with a set of normal playing cards and were asked to sort them according to the experimenter's instructions. The specific sorting tasks differed, however, for the primary school age children from the older participants. The exact instructions and procedure for each are given in Appendix B. The youngest 3 age groups of children were given a simpler dimensional change card sort task, also described in Appendix B. In each case, participants were asked to sort the cards according to one criterion first, and then according to another criterion on a second (and in the case of older participants, additional) sort. Participants' accuracy and reaction times were recorded for every sort.

### Results

#### Cost

The “cost” associated with switching from one criterion to another in the sorting tasks was measured by the difference in performance between the first and second sorts (first minus second). Both the difference scores for accuracy and for reaction times were examined.

#### Accuracy

The difference scores for accuracy were entered into an ANOVA in which age and home language were entered as variables, with the difference score as the dependent measure. There was a main effect of age group, *F*_(6, 683)_ = 4.83, *p* < 0.001, with teens performing significantly better than the 3- and 4-year-olds, Scheffe's multiple comparisons, *p*s = 0.002.

**Figure 1:**
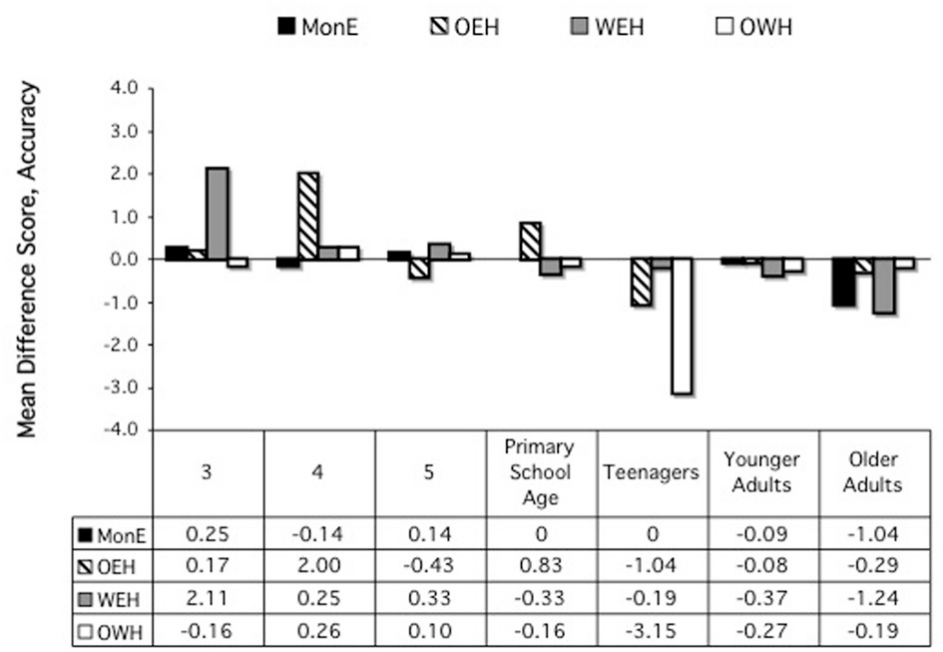
**Differences scores, accuracy, on card sort tasks, by age and home language**.

There was also a significant interaction of Age Group × Home Language, *F*_(18, 683)_ = 2.21, *p* = 0.003. Performance is shown in Figure 1. Follow-up analyses at each age revealed that there was a difference by home language only for the teen group, *F*_(3, 105)_ = 6.76, *p* < 0.001. Scheffe's multiple comparisons showed that the OWH group outperformed (i.e., had less of a switch cost than) the MonE and the WEH groups, *p*'s = 0.005, 0.004, respectively.

#### Reaction times

**Figure 2:**
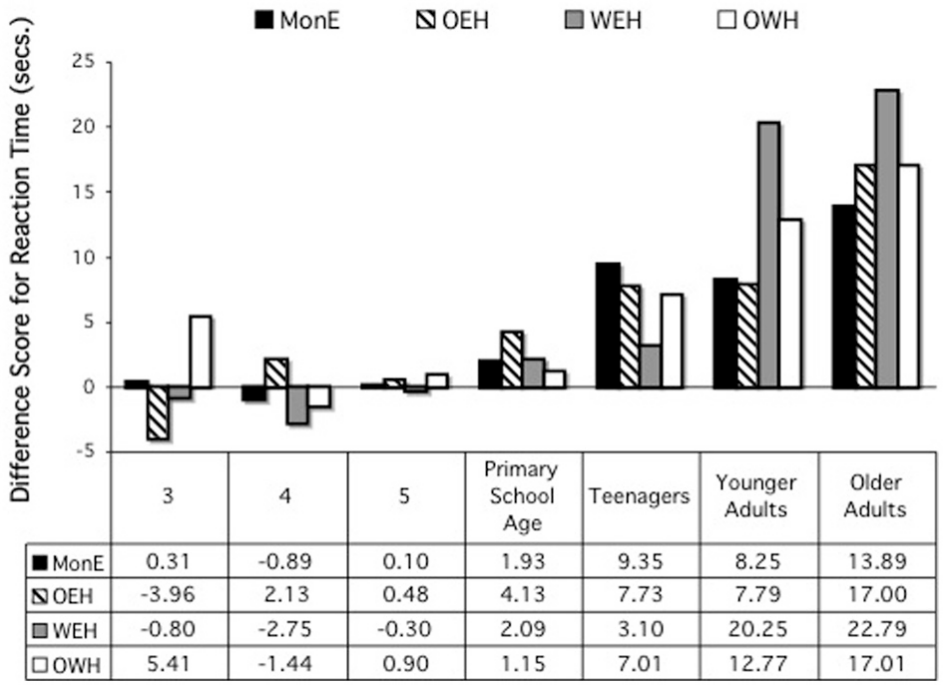
**Differences scores, reaction times, on card sort tasks, by age and home language**.

A second ANOVA examined the difference scores for reaction times. Again, age group and home language were entered as variables. This analysis revealed only a significant main effect of age group, *F*_(6, 674)_ = 25.66, *p* < 0.001. Scheffe's multiple comparisons revealed primarily differences between the two adult groups and the children's groups, with the older adults differing from all the children groups, all *p*'s < 0.001, and the younger adults from all children groups except the teens, all *p*'s < 0.001. The teens also differed significantly from the 4-year-olds, *p* = 0.033. There were no differences by home language (See Figure 2).

#### Global advantage?

To check for a possible global (BEPA) advantage for bilinguals, the data were reanalyzed, with separate tests conducted on the scores for accuracy and RTs on the first vs. second sorts by each age group. For accuracy, the only significant group effects were at the teen age group, which showed a significant HL effect, *F*_(3, 102)_ = 5.94, *p* = 0.001, and an interaction of HL × Sort, *F*_(3, 102)_ = 6.76, *p* < 0.001. These effects were due to the OWH group performing worse (at 46.6 correct) than all others (at 50.24–51.75 correct) on the first sort, *F*_(3, 120)_ = 11.15, *p* < 0.001, Scheffe's multiple comparisons, *p*s < 0.002.

For RTs, there were significant effects of HL group at ages 3 [*F*_(3, 71)_ = 3.12, *p* = 0.031], 4 [*F*_(3, 83)_ = 5.43, *p* = 0.002], 5 [*F*_(3, 79)_ = 2.95, *p* = 0.038], the teens [*F*_(3, 102)_ = 12.46, *p* < 0.001], and the younger adults [*F*_(3, 115)_ = 4.61, *p* = 0.004]. For the younger adults, there was also an interaction of HL × Sort, *F*_(3, 115)_ = 5.60, *p* = 0.001. In every case except for the teens, the Mons or English-dominant bilinguals were faster than one or more groups of the more balanced or Welsh-dominant bilinguals: at 3, Mon (34.91) < OWH (63.45), *p* = 0.057; at 4, Mon (21.68) < WEH (32.98), OWH (32.29), *p*s = 0.009, 0.022, respectively; at 5, OEH (18.73) tended to be faster than WEH (24.88), *p* = 0.062; at adults, Mon (45.07) < OWH (60.45), *p* = 0.007, Scheffe's multiple comparisons. For the teens, the Mon group was slower (62.51) than all the bilingual groups (32.83–43.50), *p*s < 0.005.

### Discussion, card sort

The results for the difference scores on the card sort tasks reveal little support for a bilingual advantage in relation to control (BICA), either for accuracy or reaction times, in relation to the costs related to a switch in the criteria to be followed in sorting. There was a single case, for accuracy at the teen years, in which the OWH children performed better than the MonE and WEH children; for RTs, MonE outperformed one or more bilingual group at ages 3, 4, 5, and younger adults; only among the teens were the bilinguals faster than the MonE children.

Similarly, the results on the absolute scores for accuracy and RTs on the first vs. second sort fail to support a global (BEPA) bilingual advantage. There was no difference by group at primary school age or among older adults; for 3-, 4-, and 5-year-olds and younger adults, the MonE or OEH participants outperformed the WEH and/or OWH participants; and for teens, the OWH group had lower accuracy rates than everyone else, but for RTs, this is the one place in which bilinguals outperformed monolinguals.

## Simon task

Two versions of the Simon Task, first created by Simon and Wolf (1963), were used in this study. We created one version specifically for younger children, and another for use with older children and adults.

### Participants

The participants for the Simon tasks were distributed as in Table 2. The mean ages are shown in Appendix A.

**Table 2:** **Participants, Simon tasks**.

**Age group**	**MonE**	**OEH**	**WEH**	**OWH**	**TOT**
3	11	20	17	22	70
4	29	9	13	16	67
5	20	16	19	16	71
Primary schoolers	13	20	17	14	64
Teens	20	28	31	35	114
Younger adults	20	19	23	23	85
Older adults	20	23	17	24	84
Total	134	136	137	150	557

### Stimuli

#### Adult version

The adult version of the task involved a blue and a red square, which appeared either on the right or the left side of the computer screen. The participant's task was to press the Q on the computer if the blue square appeared and a P if the red square appeared.

#### Child version

The child version of the task involved a rabbit and a pig, who appeared sitting on top of a rock either on the right or the left side of the computer screen. The child's task was to touch a “button” on a touch screen, to indicate whether the rabbit or the pig appeared. The “buttons” showed either the rabbit or the pig, and the rabbit button always appeared at the bottom left of the screen and the pig button always appeared at the bottom right of the screen.

### Procedure

Participants were told, both verbally and in writing on the screen, to respond as quickly as possible to indicate which item appeared. If the blue square/rabbit appeared, the Q or the button on the left was to be pressed, and if the red square/pig appeared, the P or the button on the right was to be pressed. Between trials a “+” appeared in the center of the screen. The target item appeared on the screen half of the time on the left, and half the time on the right: in “congruent” trials, the target item appeared on the same side of the screen as the key or button to be pressed; in “incongruent” trials, the item appeared on the side of the screen opposite to that on which the key or button to be pressed was located. Three practice trials were given first, and then the target trials.

School age children and adults received 48 trials, 24 congruent, and 24 incongruent, in random order. The younger children received 16 trials, 8 congruent, and 8 incongruent. Accuracy of responses and reaction times were recorded electronically.

### Results

#### Youngest ages

***Accuracy***. An ANOVA was conducted in which condition (congruent, incongruent), age group, and home language were entered as independent variables and number correct responses as the dependent variable. There were main effects of condition, *F*_(1, 196)_ = 27.25, *p* < 0.000, and of age group, *F*_(2, 196)_ = 41.27, *p* < 0.000, and an interaction of Condition × Age Group, *F*_(2, 196)_ = 9.29, *p* < 0.000. Children were more accurate in the congruent condition, with a mean of 7.07 correct, than in the incongruent condition, 6.37 correct, and performance increased between age 3, on the one hand, and 4 and 5 on the other, *p*s < 0.000, with means of 5.53, 7.20, and 7.43 at ages 3, 4, and 5, respectively.

**Figure 3:**
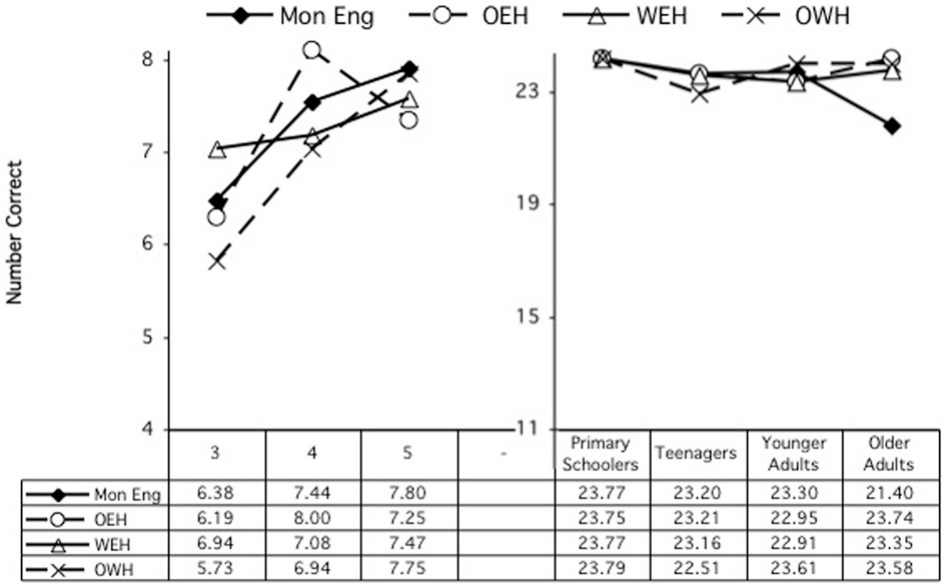
**Simon task: accuracy by age and home language, congruent condition**.

**Figure 4:**
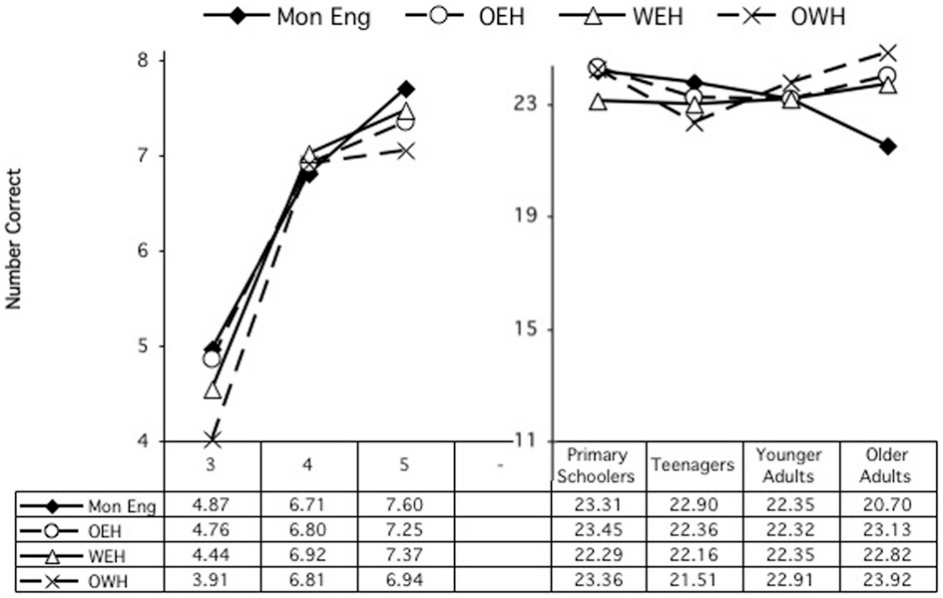
**Simon task: accuracy by age and home language, incongruent condition**.

Performance by each group is shown to the left in Figures 3, 4, showing the congruent and incongruent conditions, respectively. Follow up ANOVAs to examine the Condition × Age Group interaction looked at each age group separately. These revealed significant effects of condition (better on congruent) at ages 3 and 5, *F*_(1, 66)_ = 22.65, *p* < 0.000, and *F*_(1, 67)_ = 6.88, *p* = 0.011, respectively, but not at age 4.

There were no significant effects based on home language.

**Figure 5:**
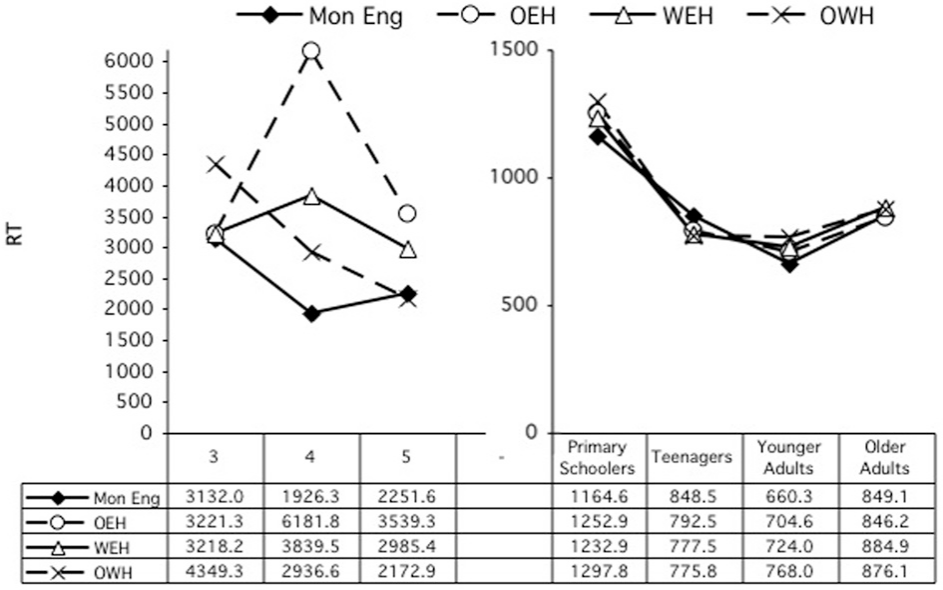
**Simon task: RT by age and home language, congruent condition**.

**Figure 6:**
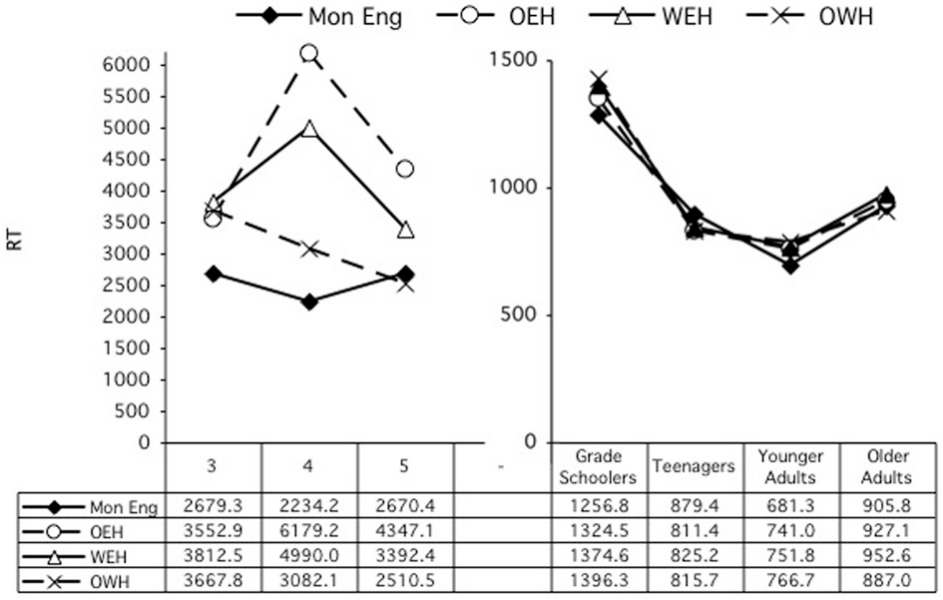
**Simon task: RT by age and home language, incongruent condition**.

***RTs***. Similarly, an ANOVA was conducted involving the same independent variables to examine reaction time performance. This analysis revealed a main effect of condition, *F*_(1, 198)_ = 4.02, *p* = 0.046, and of home language, *F*_(3, 198)_ = 3.41, *p* = 0.019. The children were generally faster in the congruent condition, 3323.29 ms, than in the incongruent condition, 3590.3 ms. Mons were significantly faster overall, at 2482.1 ms, than OEH children, 4502.05 ms, *p* = 0.002, and nearly significantly than WEH children, 3707.3 ms, *p* = 0.055; OWH children were also significantly faster (3135.69 ms) than OEH children, *p* = 0.040. There were no other main or interaction effects. Performance in the congruent and incongruent conditions are shown to the left in Figures 5, 6, which show the congruent and incongruent conditions, respectively.

#### School age children and above

***Accuracy***. An ANOVA was conducted in which condition (congruent, incongruent), age group, and home language were entered as independent variables and number correct as the dependent variable. There were main effects of condition, *F*_(1, 331)_ = 39.81, *p* < 0.000, and of age group, *F*_(3, 331)_ = 2.73, *p* = 0.044. Participants were generally more accurate in the congruent condition (23.25) than in the incongruent condition (22.58). And school-age children were more accurate than the other age groups (school-age: 23.44; teens: 22.63; younger adults: 22.84; older adults: 22.75), *p*s < 0.05.

There was also an interaction of Age Group × Home Language, *F*_(9, 331)_ = 3.14, *p* = 0.001. To explore this interaction, ANOVAs were conducted for each age group separately. Performance by each group is shown to the right in Figures 3, 4 (with the scale calibrated to show performance relative to that of the preschoolers). For the Primary Schoolers, there was no significant effect. For the teenagers, there were main effects of condition, *F*_(1, 110)_ = 18.72, *p* < 0.000, with better performance in the congruent condition (congruent: 23.02, incongruent: 22.23), and a trend in differences in performance by home language, *F*_(3, 110)_ = 2.08, *p* = 0.107. Pairwise comparisons revealed more accurate performance by the MonE participants (23.05) than the OWH participants (22.01), *p* =0.027 (with OEH and WEH in between, at 22.79 and 22.66 correct, respectively). For the younger adults, there was a main effect of condition, *F*_(1, 81)_ = 19.63, *p* < 0.000 (congruent: 23.29, incongruent: 22.48), but no effects involving home language. For the older adults, there were significant main effects of condition, *F*_(1, 80)_ = 23.72, *p* < 0.000, and of home language, *F*_(3, 80)_ = 6.12, *p* = 0.001. There was better performance on the congruent (23.02) than on the incongruent condition (22.49), and MonE participants (21.05) performed less well than all other groups, *p*s = 0.004 (OEH: 23.44, WEH: 23.09, OWH: 23.44).

***Reaction times***. Similarly, an ANOVA was conducted involving the same independent variables to examine reaction time performance. This analysis revealed a main effect of condition, *F*_(1, 330)_ = 64.98, *p* < 0.001, and of age group, *F*_(3, 330)_ = 144.42, *p* < 0.001. Participants were generally faster in the congruent condition, 903.481 ms, than in the incongruent condition, 956.080 ms. And all age groups had significantly different reaction times, all *p*s = 0.003, with the school age children the slowest (1287.55 ms), the young adults the fastest (724.72 ms), and the teens (815.75 ms) and older adults (891.11 ms) in between. There was also a significant interaction of Condition × Age Group, *F*_(3, 330)_ = 6.22, *p* < 0.000. There were no other main or interaction effects. Performance is shown to the right in Figures 5, 6 (with the scale adjusted in comparison with that for the preschoolers).

To explore the interaction of Condition × Age Group, separate ANOVAs were computed for each age group. Every age group showed faster performance on the congruent condition than on the incongruent condition: primary school age: *F*_(1, 59)_ = 32.74, *p* < 0.000; teens: *F*_(1, 110)_ = 17.97, *p* < 0.000; younger adults: *F*_(1, 81)_ = 4.41, *p* = 0.039; older adults: *F*_(1, 80)_ = 9.90, *p* = 0.002. The only group that showed an effect of home language was the younger adults, *F*_(3, 81)_ = 3.47, *p* = 0.020. In that group, the MonE participants were significantly faster overall (670.80) than both the WEH (737.94) and the OWH participants (767.35), *p*s = 0.031, 0.002, respectively.

### Summary, simon task

The results across the Simon tasks revealed that, consistent with predictions, all groups performed better on the congruent condition of the task than on the incongruent condition, but, inconsistent with predictions, there was little evidence of a bilingual advantage, either in accuracy of performance or in reaction times. Where there were effects involving home language, they were mixed. The MonE group often performed better or faster than one or more bilingual groups (for RTs in preschoolers and younger adults, for accuracy in teens); however, in one case the MonE group performed worse than the bilinguals (i.e., for accuracy among the older adults), and in another, the OWH participants patterned with the MonE participants in having faster RTs than OEH participants, in the preschool groups.

## Metalinguistic task

### Method

#### Participants

For the metalinguistic task, a total of 354 participants were tested, from four age groups: primary schoolers, teens, younger adults, and older adults. The distribution of participants by age group and home language was as shown in Table 3. The Monolingual English participants were given only the English task; all three bilingual home language groups were given both the English and the Welsh task.

**Table 3:** **Participants, metalinguistic task, English and Welsh**.

**Age group**	**MonE**	**OEH**	**WEH**	**OWH**	**TOT**
Primary schoolers	12	19	15	21	67
Teens	21	23	24	35	103
Younger adults	27	21	24	25	97
Older adults	22	21	17	27	87
Total	82	84	80	108	354

The mean ages for each group are shown in Appendix A.

#### Stimuli

For both languages, 24 sentences were drawn up. In these, 6 types of structures were manipulated, and each type of structure was used in a grammatical meaningful sentence (“GM”), a grammatical, but anomalous sentence (“Gm”), an ungrammatical meaningful sentence (“gM”), and an ungrammatical anomalous sentence (“gm”). The 6 types of structures involved subject-verb agreement, irregular past tense formation, position of object pronouns, subject-auxiliary inversion in *wh-* questions, co-occurrence restrictions between the comparative form and the standard marker (*than*), and sequence of tenses. This design yielded 6 trials for each of the sentential conditions, GM, Gm, gM, and gm.

Examples of the English and Welsh sentences involving subject-verb agreement are shown in (1) in Table 4, and involving irregular past tense are shown in (2) in Table 4.

**Table 4:** **Sample sentential stimuli for the metalinguistic task**.

	**GM**	**Gm**	**gM**	**gm**
**(1) SUBJECT-VERB AGREEMENT**				
E	Today, Tommy is travelling to the zoo to visit the animals.	Today, Jenny is walking to the park to play with clouds.	Today, Billy am riding to the airport to see the planes.	Today, Mary are going to the supermarket to buy three pilots.
W	Heddiw, mae Tomi yn teithio i'r sw i weld yr anifeiliaid.	Heddiw, mae Jini yn cerdded i'r parc i chwarae ar y cymylau.	Heddiw, rydw Bili yn rhedeg i'r maes awyr i weld yr awyrennau. [requires *mae*, not *rydw*]	Heddiw, roeddwn Mari yn mynd i'r siop i brynnu tri peilot. [requires *roedd*, not *roeddwn*]
**(2) IRREGULAR PAST TENSE**				
E	Sam finished his work, so he gave his paper to the teacher to mark.	Sue ate her lunch, so she left her plate for the cook to break.	Jim did his painting, so he bringed his brush to his dad to clean.	Jan read the story, so she taked the book to the librarian to chew.
W	Gorffennod Sam ei waith, felly mi roddodd ei bapur i'r athrawes i'w farcio.	Bwytaodd Siwan ei chinio, felly gadawodd ei phlat i'r ddynes cinio dorri.	Peintiodd Jim ei lun, felly daethodd â'r brws i'w dad i olchi. [requires *daeth*, not *daethodd*]	Darllennodd Sian y stori, felly aethwyd hi â'r llyfr i'r llyfrgellydd i gnoi. [requires *aeth*, not *aethwyd*]

For the two languages, two versions of the sentences were drawn up. In the two versions, items that were grammatical and/or meaningful in one appeared as ungrammatical and/or anomalous in the other. For example, in one version, “Jim did his painting, so he bringed his brush to his dad to clean” occurred as gM, and in the other “Jim did his painting, so he bringed his brush to his dad to wear” occurred as gm. Bilingual participants heard the English sentences from one of these versions and Welsh sentences from the other. Monolinguals heard the English sentences from only one of the versions. The use of the two versions in each language across the participants was balanced.

#### Procedure

Participants heard sentences read to them orally. They were asked to judge whether a sentence was grammatical, and to correct it if it was ungrammatical. (See Appendix C for more details.) Participants were given 5 practice sentences, and then the target trials. The trial sentences were given in random order.

### Results

#### English

An ANOVA was conducted in which condition (GM, Gm, gM, gm), age, and home language were entered as independent variables and number correct responses as the dependent variable. There were significant main effects for all variables: condition, *F*_(3, 1014)_ = 128.81, *p* < 0.000; age, *F*_(3, 338)_ = 63.55, *p* < 0.000; home language, *F*_(3, 338)_ = 2.99, *p* = 0.031. Overall, participants performed differently on all conditions, pairwise comparisons, *p*s = 0.001, with performance best for GM (5.60 correct), next best for Gm (5.19), next for gM (4.88), and least good for gm (3.95). Similarly, all age groups performed significantly differently, all *p*s = 0.015, with improvement with age: primary schoolers: 3.79, teens: 4.92, younger adults: 5.30, older adults: 5.61. The effect of home language was due to significantly better performance overall by the OEH participants (5.11) over the WEH (4.73) and OWH (4.84) participants, pairwise *p*s = 0.005, 0.027, respectively. (MonE fell between the two extremes: 4.93).

These main effects were modified by a significant interaction of Condition × Age, *F*_(9, 1014)_ = 8.31, *p* < 0.000, a near-significant effect of Condition × Home Language, *F*_(9, 1014)_ = 1.84, *p* = 0.058, and a significant interaction of Condition × Age × Home Language, *F*_(27, 1014)_ = 1.55, *p* = 0.036. To examine the interactions, performance by each age group was analyzed separately.

**Figure 7:**
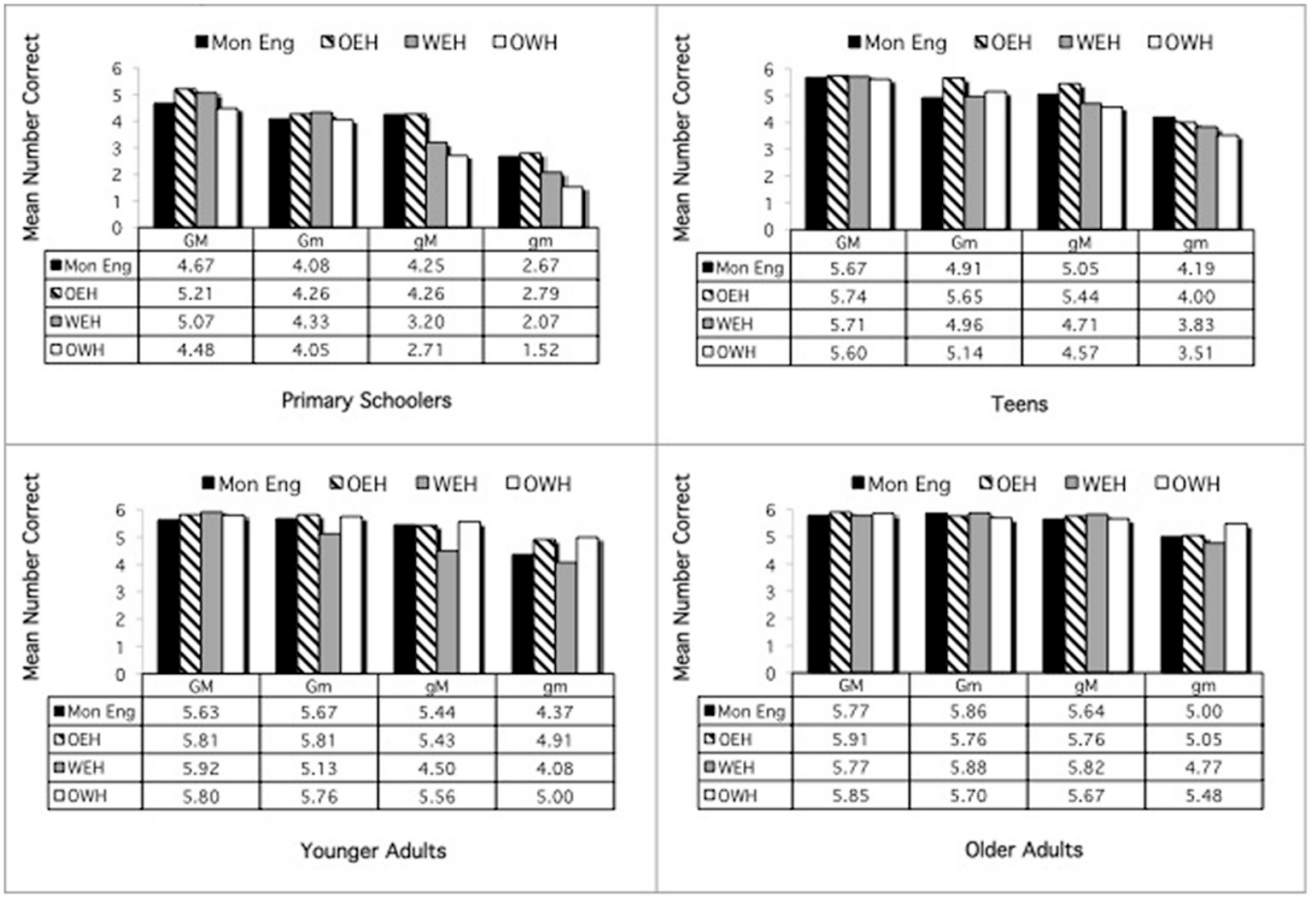
**Metalinguistic task: English**.

Performance at each age is shown in Figure 7. The primary schoolers showed a significant main effect of condition, *F*_(3, 189)_ = 40.75, *p* < 0.000. Performance on all conditions was significantly different, *p*s < 0.000, except for the Gm and gM conditions, which reached near-significance, *p* = 0.073. There was a near-significant interaction of Condition × Home Language, *F*_(9, 189)_ = 1.83, *p* = 0.066. Follow-up analysis revealed a significant difference in performance on the gM sentences, *F*_(3, 63)_ = 3.42, *p* = 0.022, with OWH children performing lower than the OEH children, *p* = 0.044. The teens likewise showed a significant effect of condition, *F*_(3, 297)_ = 38.52, *p* < 0.000, with significant differences across all conditions, *p*s = 0.001, except for the Gm and gM conditions, *p* = 0.238. There were no other differences among the teens. The younger adults also showed an effect of condition, *F*_(3, 279)_ = 24.48, *p* < 0.000, with all conditions significantly different, *p*s = 0.036, except for the GM and Gm sentences, which were nearly significantly different, *p* = 0.068. There were no other differences among the younger adults. The older adults likewise showed a significant effect of condition, *F*_(3, 249)_ = 21.29, *p* < 0.001, but here performance differed only on the gm condition relative to all the others, *p*s < 0.000. There were no other differences.

#### Welsh

An ANOVA was similarly conducted examining performance on the Welsh sentences. There were significant main effects for all variables: condition, *F*_(3, 780)_ = 169.56, *p* < 0.001; age, *F*_(3, 260)_ = 56.80, *p* < 0.001; home language, *F*_(2, 260)_ = 3.90, *p* = 0.021. Overall, participants performed differently on all conditions, pairwise comparisons, *p*s = 0.000, with performance best for GM (5.56 correct), next best for Gm (5.10), next for gM (4.14), and least good for gm (3.52). Similarly, most age groups performed significantly differently, all *p*s = 0.001, except for the younger and older adults, who did not differ significantly, *p* = 0.144. Performance improved with age: primary schoolers: 3.32, teens: 4.59, younger adults: 5.09, older adults: 5.31. The effect of home language was due to significantly better performance overall by the OWH participants (4.79) and the WEH participants (4.50) over the OEH participants (4.45), pairwise *p*s = 0.033, 0.012, respectively.

These main effects were modified by a significant interaction of Condition × Age, *F*_(9, 780)_ = 12.12, *p* < 0.000, and of Condition × Home Language, *F*_(6, 780)_ = 3.03, *p* = 0.006. There were no other interactions. Follow-up analyses examined these interactions.

**Figure 8:**
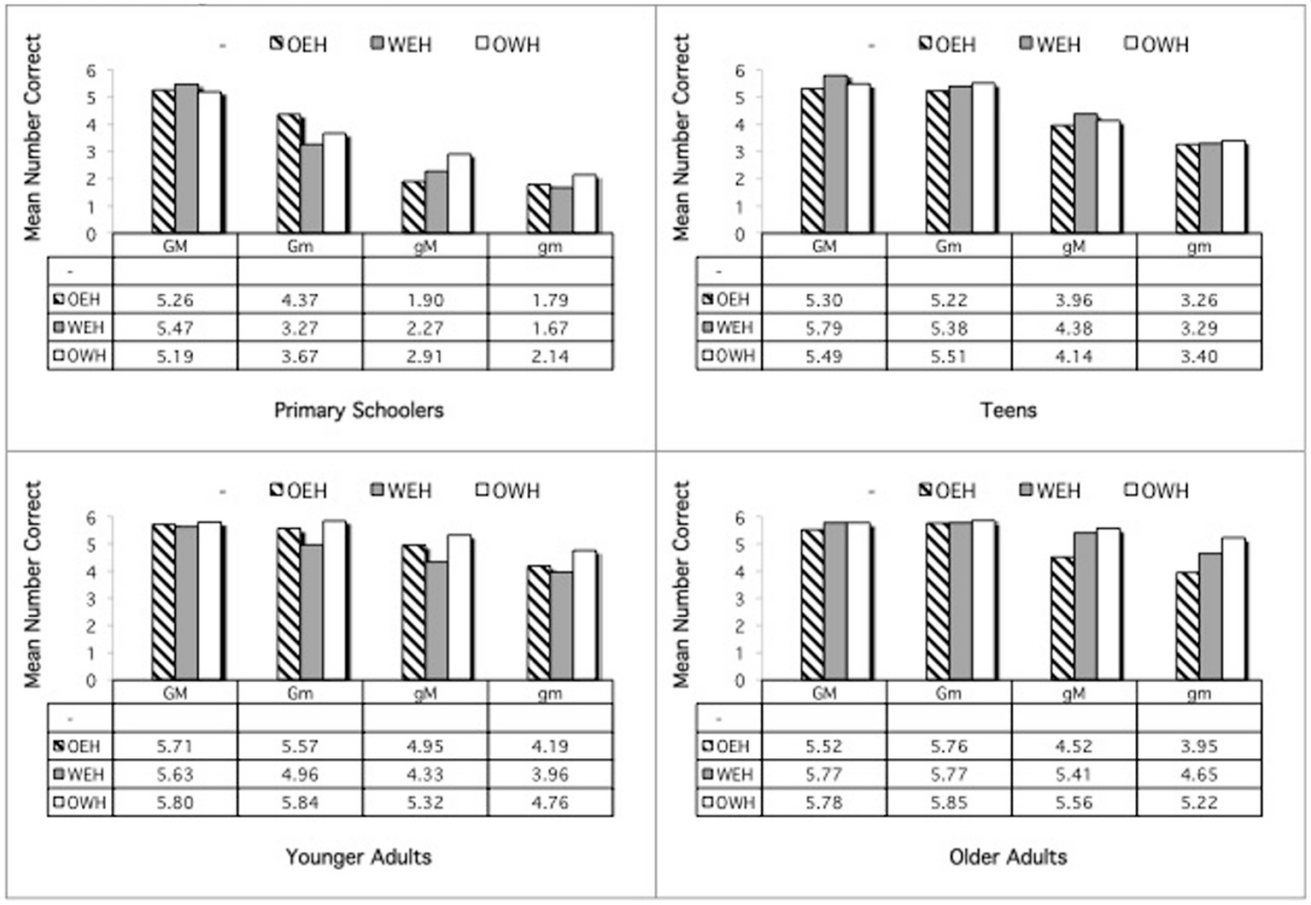
**Metalinguistic task: Welsh**.

Performance at each age is shown in Figure 8. First, each age group was examined separately to explore the Condition × Age interaction. Analyses revealed that the Condition × Age interaction reflects the fact that performance differed on all conditions for the primary schoolers, pairwise *p*s = 0.024, but for the other age groups, all but the GM vs. Gm conditions differed, pairwise *p*s = 0.002. The Condition × Home Language interaction was explored by examining each condition separately. Analysis revealed that performance differed by home language only on the gM condition, *F*_(2, 269)_ = 2.95, *p* = 0.054. The OWH participants performed significantly better here (4.53) than the OEH participants (3.88), *p* = 0.049.

### Summary, metalinguistic task

The results of the metalinguistic tasks also failed to reveal a bilingual advantage, either overall or in the crucial Gm condition, which requires the greatest levels of inhibitory control. This is contrary to expectations, according to the proposal of an executive function advantage by bilinguals in this condition. In accordance with predictions related to language ability, in contrast, home language, when it mattered, showed an advantage in the direction of the bilingual group that was dominant in the given language. That is, for English, the OEH children performed the best of the bilinguals; and in the primary age group, on the gM condition (which requires greater levels of sentence analysis than control of attention), the OEH children outperformed the OWH children; in contrast in Welsh, the OWH and WEH participants outperformed the OEH participants, and specifically in the gM condition, the OWH participants outperformed the OEH participants.

## Discussion

These experiments reveal that on three sets of executive function tasks, performance by this group of simultaneous and early sequential bilinguals fails to provide support for an overall bilingual advantage at any of the seven ages tested here. The card sorting tasks failed to show an overall advantage of bilinguals, either in relation to the “cost” of the switch or in relation to an overall performance advantage. On the Simon task, performance was generally similar across groups, or the monolinguals generally had the advantage; in many cases, the monolinguals (or in one case, the OEH bilinguals) were faster or more accurate than one or more groups of bilinguals. In one case, however, the OWH bilinguals were, like the monolinguals, also faster than the OEH and WEH bilinguals, and in one case, the monolinguals were less accurate than the bilinguals (at the older adult group). On the metalinguistic task, again where there were differences, the differences were in the direction of those dominant in the language being tested outperforming those who were less dominant, most importantly, even in the Gm condition, where executive control was predicted to favor bilinguals.

It should be noted that this evidence showing little support for the bilingual advantage was accompanied in every case by robust evidence supporting predictions not related to home language. For example, performance in congruent conditions was always superior to performance in incongruent conditions, both in accuracy and in RTs (similar to findings in Kousaie and Phillips, 2012; Paap and Greenberg, 2013; Duñabeitia et al., 2013); changes with age in children always showed better performance with age; changes with age in adults often showed decreased performance at the older ages; judgments of grammaticality were better with grammatical sentences than with ungrammatical sentences. This indicates that the tasks here elicited performance as predicted in all major ways except for one, in relation to bilingualism.

The absence of strong support for the position of a bilingual advantage on these executive tasks, as in our earlier work (and in some forthcoming work from Clare et al., submitted) is striking. This study examined a large number of fully fluent, simultaneous and early sequential bilinguals, homogeneous in cultural and educational backgrounds, and homogeneous with those of the monolinguals. While it is possible that language abilities contributed to performance on the card sorting and metalinguistic tasks, the Simon task is a classic task used to examine EF performance. The results here suggest that whatever mechanisms yield superior performance in other studies in relation to bilinguals and control may be less relevant to simultaneous and early sequential bilinguals.

As noted above, in many studies, the participants are L2 bilinguals (or not clearly defined, Adesope et al., 2010). The process of acquiring two languages and the relationship between the bilingual's two languages are clearly different in simultaneous bilinguals than in L2 bilinguals (see, e.g., Li, 2010), and one can predict that the use of language in the former group is likely to be more automatic and less effortful than in the latter group. This may make the theoretical issues surrounding control in bilinguals less relevant to simultaneous bilinguals than to L2 bilinguals. Paap and Greenberg (2013) point out that one of the background assumptions for theories of a bilingual advantage in EF is that “the amount of EP recruited by bilinguals during language comprehension and production is greater than that employed by monolinguals” (p. 255), but that speaking any language, whether bilingually or monolingually, involves a great deal of monitoring, switching, and inhibitory control. They add:

To provide just a few examples, conversational participants must monitor the environment for signals regarding turn-taking, misunderstandings, possible use of sarcasm, changes of topic, or changes in register contingent upon who enters or leaves the conversation. These lead to switches from speaker to listener, switches from one knowledge domain to another, and so forth. Although monolinguals do not need to suppress translation equivalents during production, they incessantly make word choices among semantically and syntactically activated candidates that include synonyms, hypernyms, and hyponyms. In addition monolinguals must use context to suppress irrelevant meaning of homographs during comprehension (p. 256).

It is worth considering as well the extent to which the theory surrounding a bilingual advantage in relation to control hinges on a modular approach to language. If the two languages spoken by a bilingual are separate, then this would necessarily involve some mechanism for switching back and forth between the two languages. Consider, however, a less modular model of language. Under a computational model of language acquisition and language use, for example, the processes involved in language use can be seen more as involving activation of links than switches between two separate (but related) systems. The links within a language will be stronger than across languages, but both languages appear to be “on line” at all moments (see, e.g., Lam and Dijkstra, 2010). In fully fluent simultaneous bilinguals, in contrast to, e.g., recent or less fluent L2 language learners, the automaticity of their linguistic knowledge in both languages may mean that whatever “switching” they are carrying out is a function of the contexts of speech, just as it is for monolinguals. Less fluent bilinguals, L2 learners, on the other hand, may need to conduct a greater level of control in every linguistic choice they make. It is striking that much of the literature in which no bilingual advantage has been found has involved fully fluent bilingual communities such as this one in North Wales and the Basque Country (e.g., Duñabeitia et al., 2013).

These questions are deserving of much closer scrutiny in future research. The choice of participants in studies of this type needs to be controlled more carefully in the future, so that we can better define exactly who shows an advantage in performance, under what conditions, and why.

### Conflict of interest statement

The authors declare that the research was conducted in the absence of any commercial or financial relationships that could be construed as a potential conflict of interest.

## Footnotes

^1^A child was classified as OWH (OEH) if the parents reported at least 80% use of Welsh (English) in the home in speech to the child from birth to the present time, and adults were classified as OWH (OEH) according to the “origin home language,” the home language patterns in their homes when they were children. Participants were classified as WEH if they received between 40 and 60% use of both languages in the home from their parents.

## Appendix A

### Mean ages of participants

#### Card sort tasks

Mean ages by group were as follows:

3: mean: 3.0; range of means in the individual home language groups: 2.11–3.2 (individual range: 2.0–3.6)

4: mean: 4.2; range of means in the individual home language groups: 4.1–4.3 (individual range: 3.7–4.9)

5: mean: 5.5; range of means in the individual home language groups: 5.4–5.6 (individual range: 4.10–6.0)

Primary Schoolers: mean: 8.0; range of means in the individual home language groups: 7.8–8.5 (individual range: 7.0–8.11)

Teens: mean: 14.9, range of means in the individual home language groups: 14.7–14.11 (individual range: 13.0–16.0)

Younger Adults: mean: 24.8; range of means in the individual home language groups: 23.5–26.2 (individual range: 18.0–39.0)

Older Adults: mean: 67.4; range of means in the individual home language groups: 65.1–69.1 (individual range: 57.0–90.0)

#### Simon task

Mean ages for each group were as follows:

3: mean: 3.0; range of means in the individual home language groups: 2.11–3.3 (individual range: 2.0–3.6)

4: mean: 4.2; range of means in the individual home language groups: 4.1–4.5 (individual range: 3.5–4.11)

5: mean: 5.4; range of means in the individual home language groups: 5.4–5.6 (individual range: 4.10–6.0)

Primary School: mean: 8.2; range of means in the individual home language groups: 7.9–8.5 (individual range: 7.0–8.11)

Teens: mean: 14.9; range of means in the individual home language groups: 14.8–14.11 (individual range: 13.0–16.0)

Younger Adults: mean: 25.5; range of means in the individual home language groups: 23.4–26.6 (individual range: 18.3–39.0)

Older Adults: mean: 67.6; range of means in the individual home language groups: 66.2–68.4 (individual range: 57.6–90.0)

#### Metalinguistic task

The mean ages were as follows:

Primary School: mean: 8.2; range of means in the individual home language groups: 7.10–8.4 (individual range: 7.0–8.11)

Teens: mean: 14.10; range of means in the individual home language groups: 14.8–15.0 (individual range: 13.0–16.0)

Younger Adults: mean: 25.4; range of means in the individual home language groups: 23.2–27.2 (individual range: 18.3–39.0)

Older Adults: mean: 67.7; range of means in the individual home language groups: 66.2–68.5 (individual range: 57.6–90.0)

## Appendix B

### Method and procedures, card sort tasks

#### Primary school age children

***Materials***. A set of 24 cards was used, the 2s, 3s, 4s, 8s, 9s, and 10s from all four suits of a normal card deck. The 2s, 3s, and 4s were considered “low” numbers, the 8s, 9s, and 10s the “high” numbers. The four sorts that this group were asked to conduct involved (A) low red cards, (B) high clubs, (C) low diamonds, and (D) low black cards. A stop watch was used for timing response time.

***Procedure***. The experimenter instructed the child to sort the cards four times, into low red cards, high clubs, low diamonds, and low black cards, in that order or its reverse. Approximately half the children received the sorts in ABCD order, approximately half in DCBA order.

#### Teens, younger adults, and older adults

***Materials***. A full set of 52 cards was used. The four sorts that this group were asked to conduct involved (A) odd red cards, (B) even clubs, (C) odd diamonds, and (D) odd black cards. A stop watch was used for timing.

***Procedure***. The experimenter instructed the participant to sort the cards four times, into (A) odd red cards, (B) even clubs, (C) odd diamonds, and (D) odd black cards. Approximately half the participants received the sorts in ABCD order, approximately half in DCBA order.

#### Younger children/preschoolers

***Materials***. A set of 20 cards was used. These cards depicted two types of shapes, circles, and squares (“balls” and “blocks”), and they were of two sizes, small, and large. Children were asked to first sort the cards according to one of these features (shape or size), and then according to the other. A stop watch was used for timing.

***Procedure***. The child was asked to sort the cards twice, into balls and blocks or into big and little shapes. Approximately half the children received the ball/block sort first, approximately half the big shape/little shape sort first.

## Appendix C

### Procedure for the metalinguistic task

Participants heard sentences read to them orally. They were asked to judge whether a sentence was grammatical, and to correct it if it was ungrammatical, with the following instructions:

These are my friends, Sali and Twmi. Sometimes, Twmi gets mixed up when he talks. Sometimes, he doesn't know how to talk very well. Sometimes, he says things right but they're just silly. Sometimes, he says things that make sense, but he says them wrong. And sometimes, he says things that are silly *and* wrong. Sali helps him when he says things the wrong way. She tells Twmi how he should say it. It's okay to say silly things sometimes though, isn't it? So Sali only tells him to say things right when Twmi says them wrong. If he says something that's silly but sounds right, that's ok.

For example, if Twmi says “I am a banana,” Sali says “That's very silly but you said it right.”

And if Twmi says “My hair be yellow” then Sali says “That makes sense,” but it's not right, you should say “my hair *is* yellow”

And if Twmi says “I can talk,” Sali says “yes, that's right.”

And if Twmi says “I be a lemon,” Sali says “That's silly *and* it's wrong.” It's okay to be silly, but you should say “I *am* a lemon.”

So, if Twmi says (Practice Sentence 1), what do you think Sali will say? (Child responds Right, Silly or Not Right). How should Sali tell Twmi to say it?
